# Predictors and outcomes of postoperative tracheostomy in patients undergoing acute type A aortic dissection surgery

**DOI:** 10.1186/s12872-022-02538-4

**Published:** 2022-03-09

**Authors:** Dashuai Wang, Su Wang, Yu Song, Hongfei Wang, Anchen Zhang, Long Wu, Xiaofan Huang, Ping Ye, Xinling Du

**Affiliations:** 1 Department of Cardiovascular Surgery, Union Hospital, Tongji Medical College, Huazhong University of Science and Technology, Wuhan, 430022 China; 2 Department of Emergency Medicine, Union Hospital, Tongji Medical College, Huazhong University of Science and Technology, Wuhan, 430022 China; 3 Department of Cardiology, The Central Hospital of Wuhan, Tongji Medical College, Huazhong University of Science and Technology, Wuhan, 430014 China

**Keywords:** Acute type A aortic dissection, Postoperative tracheostomy, Risk factors, Outcomes, Nomogram

## Abstract

**Background:**

Despite surgical advances, acute type A aortic dissection remains a life-threatening disease with high mortality and morbidity. Tracheostomy is usually used for patients who need prolonged mechanical ventilation in the intensive care unit (ICU). However, data on the risk factors for requiring tracheostomy and the impact of tracheostomy on outcomes in patients after Stanford type A acute aortic dissection surgery (AADS) are limited.

**Methods:**

A retrospective single-institutional study including consecutive patients who underwent AADS between January 2016 and December 2019 was conducted. Patients who died intraoperatively were excluded. Univariate analysis and multivariate logistic regression analysis were used to identify independent risk factors for postoperative tracheostomy (POT). A nomogram to predict the probability of POT was constructed based on independent predictors and their beta-coefficients. The area under the receiver operating characteristic curve (AUC) was performed to assess the discrimination of the model. Calibration plots and the Hosmer–Lemeshow test were used to evaluate calibration. Clinical usefulness of the nomogram was assessed by decision curve analysis. Propensity score matching analysis was used to analyze the correlation between requiring tracheostomy and clinical prognosis.

**Results:**

There were 492 patients included in this study for analysis, including 55 patients (11.2%) requiring tracheostomy after AADS. Compared with patients without POT, patients with POT experienced longer ICU and hospital stay and higher mortality. Age, cerebrovascular disease history, preoperative white blood cell (WBC) count and renal insufficiency, intraoperative amount of red blood cell (RBC) transfusion and platelet transfusion were identified as independent risk factors for POT. Our constructed nomogram had good discrimination with an AUC = 0.793 (0.729–0.856). Good calibration and clinical utility were observed through the calibration and decision curves, respectively. For better clinical application, we defined four intervals that stratified patients from very low to high risk for occurrence of POT.

**Conclusions:**

Our study identified preoperative and intraoperative risk factors for POT and found that requiring tracheostomy was related to the poor outcomes in patients undergoing AADS. The established prediction model was validated with well predictive performance and clinical utility, and it may be useful for individual risk assessment and early clinical decision-making to reduce the incidence of tracheostomy.

## Introduction

Respiratory failure needing prolonged ventilator support is a frequent and serious complication and is also a common cause of in-hospital deaths in patients undergoing Stanford type A acute aortic dissection surgery (AADS) [1]. About 28.9%-34.6% of patients with AADS had prolonged mechanical ventilation postoperatively, which was associated with an increased risk of morbidity and mortality [2, 3]. Tracheostomy is commonly used for patients requiring prolonged mechanical ventilation in the intensive care unit (ICU) to optimize the work of breathing or facilitate weaning [4]. Some studies reported the number of patients undergoing tracheostomy after AADS and the incidence was about 4.7% to 11.8% [2, 5, 6].

Patients who underwent tracheostomy had been found to have significantly longer mechanical ventilation time and hospital stay but a lower hospital mortality compared with those not receiving a tracheostomy among the patients requiring mechanical ventilation in the ICU setting [7]. In addition, the dramatically longer duration of hospitalization and worse survival were observed in patients requiring tracheostomy after some major surgeries, such as liver transplant and congenital heart surgery [8, 9]. However, the outcomes of patients receiving a tracheostomy after AADS have rarely been described before. Moreover, to our knowledge, no studies have been conducted to identify clinical risk factors of postoperative tracheostomy (POT) in patients after AADS.

The present study was undertaken to explore the relationship between POT and the outcomes in patients who underwent AADS, and also to identify independent predictors for POT among these patients and thus construct a prediction model to predict the occurrence of POT.

## Methods

### Study population

A retrospective study was performed at a single center. The consecutive patients who underwent AADS at our institution between January 2016 and December 2019 were studied. Patients who died during the surgery were excluded from this study.

This study was conducted in accordance with ethical statement of the Declaration of Helsinki and was approved by The Ethics Committee of Tongji Medical College of Huazhong University of Science and Technology (IORG No. IORG0003571). The requirement of written informed consent was waived by The Ethics Committee of Tongji Medical College of Huazhong University of Science and Technology (IORG No. IORG0003571) due to its observational, retrospective nature.

### Baseline data

Baseline data including demographics, comorbidities, preoperative laboratory values and surgical procedure were documented. Specifically, the demographics contained age, sex, body mass index, history of smoking and drinking; comorbidities included diabetes mellitus, hypertension, cardiac surgery history, general surgery history, pulmonary artery hypertension, cerebrovascular disease, atrial fibrillation, pericardial effusion, chronic obstructive pulmonary disease, left ventricular ejection fraction, gastrointestinal tract disease, NYHA class III-IV, peripheral vascular disease, renal insufficiency, diameter of the left ventricle, diameter of the right ventricle, diameter of the right atrium, and diameter of the left atrium; preoperative laboratory data contained platelet count, serum albumin, serum globulin, serum creatinine, white blood cell (WBC) count, red blood cell (RBC) count, hemoglobin; surgical procedure included isolated AADS, AADS combined with coronary artery bypass grafting, AADS combined with coronary artery bypass grafting, AADS combined with other types of cardiac surgery, AADS combined with valve surgery, AADS combined with valve and coronary surgery, cardiopulmonary bypass time, deep hypothermic circulatory arrest, aortic cross clamp time, transfusion of red blood cells and platelet.

### Endpoints

Performing a tracheostomy on the patients after AADS was the primary endpoint of this study. The clinicians performed the tracheostomies through the percutaneous route in the ICU. The indications for tracheostomy in this study included bypass of upper airway obstruction, prolonged mechanical ventilation, predicted difficult reintubation, repeated intubation, one or more failed trails of extubation, and the need for tracheal access to remove thick pulmonary secretions. The secondary endpoints included readmission to ICU, the length of ICU and hospital stays, and mortality.

### Derivation and verification of predictive model

We used clinical data to develop a risk model to predict the probability of tracheostomy in patients after they received AADS. Univariate analysis was used to screen out variables that were possibly associated with POT in the cohort. The potential risk factors which were significant in the univariate analysis (P < 0.10) were entered into the multivariate logistic regression analysis. A nomogram was constructed based on the independent predictors and their beta-coefficients in the multivariate analysis. Internal validation of the prediction model was assessed by bootstrapping using 1000 replications. The calibration, discrimination and clinical utility were conducted to evaluate the predictive performance of the nomogram. The area under the receiver operating characteristic (ROC) curve (AUC) or Harrell’s concordance index (C-index) was performed to assess the discrimination of the prediction model. The calibration curve was used to analyze the agreement between nomogram-predicted and actually observed probability. Decision curve analysis was conducted to assess the clinical utility of the nomogram by quantifying the standardized net benefits at different risk threshold.

### Statistical analysis

SPSS software (version 26.0) and R software (version 4.0.3) were used for statistical analysis. Descriptive statistical analyses were performed for categorical and continuous variables. Categorical variables were presented as n (%), and continuous variables were presented as mean ± standard deviation or median (interquartile range). We compared categorical variables using the Chi-square or Fisher exact test, non-normal variables with the Mann–Whitney U test, normally distributed variables with the Student's t test. Propensity score matching analysis was performed to reduce potential selection bias to analyze the correlation between requiring tracheostomy and clinical prognosis in this study. The nearest available matching (1:1) method was used for propensity score matching with the caliper of 0.03. P value < 0.05 was considered statistically significant.

## Results

### Baseline characteristics

**Fig. 1:**
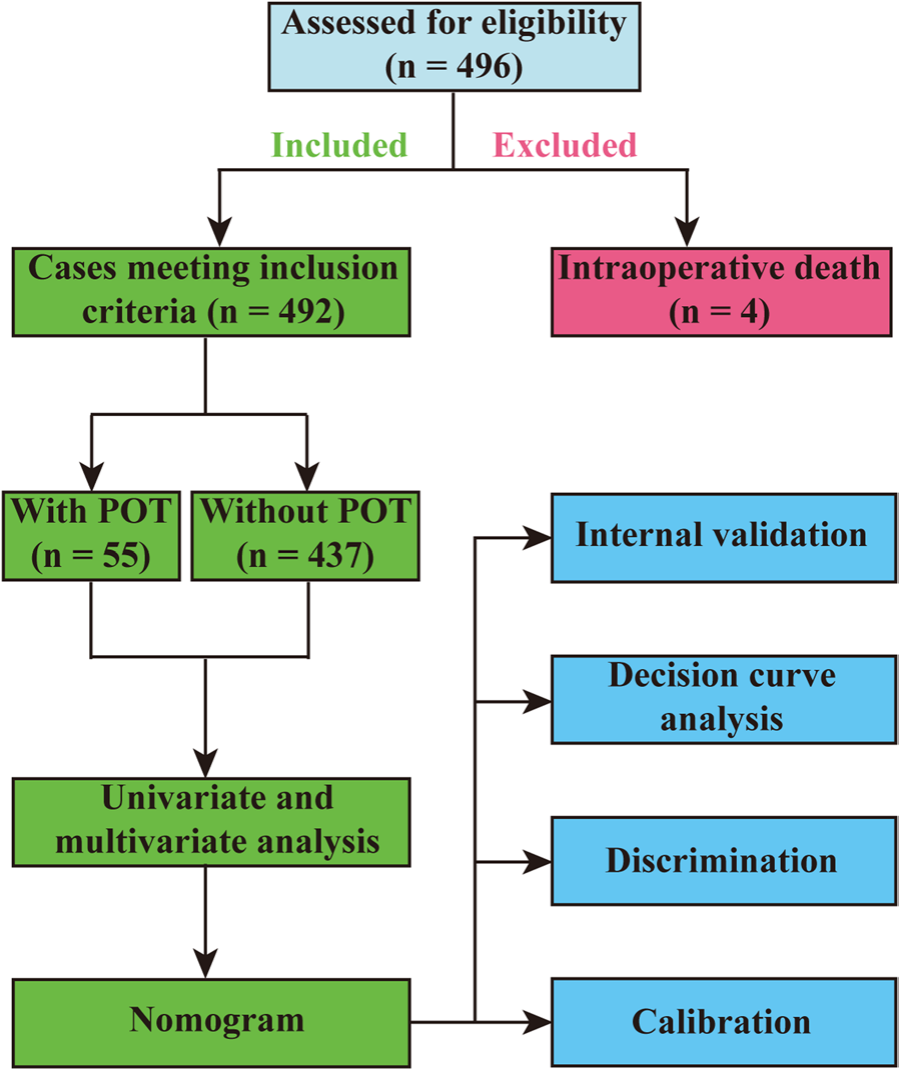
Flow chart of the study. AADS, Stanford type A acute aortic dissection surgery; POT, postoperative tracheostomy

A total of 496 patients underwent type A acute aortic dissection repair surgeries during the 4-year period in our institution. After excluding 4 patients who died intraoperatively, 492 patients were included in the analysis. Of these, 55 patients (11.2%) required tracheostomy after AADS. The flow chart of this study is presented in Fig. 1. The average age of the entire cohort was 49.6 years, males accounted for 75.6%, the average body mass index reached 25.3 kg/m^2^, and the proportion of smoking history was 43.9%. In addition, hypertension, isolated AADS, and deep hypothermic circulatory arrest application, these variables accounted for more than 50% of the population in our study.

### Development and validation of risk predictive model

**Fig. 2:**
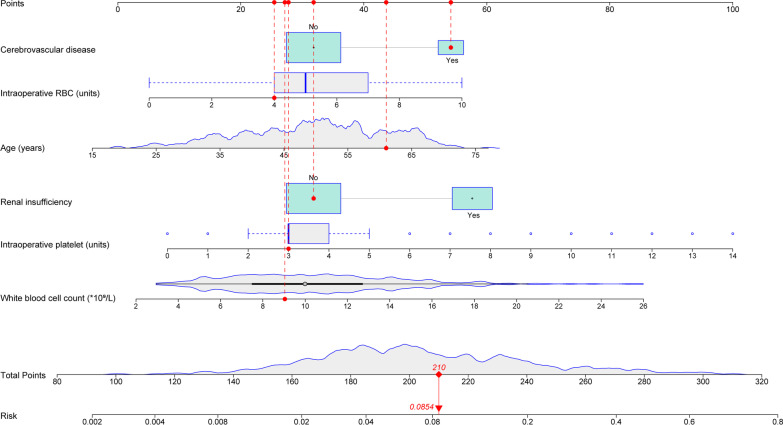
Nomogram for predicting postoperative tracheostomy. Each red dot represents each variable value of the patient. The total point is 210, corresponding to a probability of 8.5% to develop postoperative hyperlactatemia. RBC, red blood cell

The univariate analysis was performed to screen out several potential risk factors for POT in patients undergoing AADS (Table 1). Before the establishment of a multivariate model, collinearity diagnostics were conducted. By stepwise forward selection, six risk factors were identified to independently predict the occurrence of POT, including age, cerebrovascular disease history, preoperative WBC count and renal insufficiency, intraoperative amount of RBC transfusion and platelet transfusion (Table 2). Then a nomogram was constructed based on previous multivariate model, and the regression coefficient of each predictor was scaled to points of 0–100, reflecting the relative importance of each predictor (Fig. 2). The probability of needing tracheostomy in a patients can be calculated by summing all the variables scores, and the risk of POT after AADS ranged from 0.002 to 0.8 on the nomogram.

**Table 1:** Univariate analysis of possible risk factors for POT after AADS

Characteristic	Without POT n = 437 (%)	With POT n = 55 (%)	χ^2^/Z/t	*P* value
*Demographics*				
Male	322 (73.7)	50 (90.9)	7.860	0.005
Age (years)	49.27 ± 11.33	52.58 ± 10.83	2.051	0.041
Body mass index (kg/m^2^)	25.26 ± 3.65	25.93 ± 4.04	1.226	0.206
Smoking history	186 (42.6)	30 (54.5)	2.848	0.091
Drinking history	151 (34.6)	25 (45.5)	2.527	0.112
*Underlying conditions*				
Hypertension	292 (66.8)	43 (78.2)	2.903	0.088
Diabetes mellitus	19 (4.3)	2 (3.6)	0.061	0.806
Chronic obstructive pulmonary disease	5 (1.1)	0 (0)	0.636	0.425
Cerebrovascular disease	71 (16.2)	17 (30.9)	7.150	0.007
Peripheral vascular disease	63 (14.4)	4 (7.3)	2.119	0.145
Renal insufficiency	138 (31.6)	35 (63.6)	22.021	< 0.001
Gastrointestinal tract disease	37 (8.5)	5 (9.1)	0.024	0.876
Atrial fibrillation	2 (0.5)	2 (3.6)	6.121	0.013
Cardiac surgery history	29 (6.6)	3 (5.5)	0.112	0.738
General surgery history	90 (20.6)	11 (20.0)	0.011	0.918
NYHA class III-IV	38 (8.7)	3 (5.5)	0.672	0.412
Pulmonary artery hypertension	14 (3.2)	0 (0)	1.814	0.178
Pericardial effusion	121 (27.7)	12 (21.8)	0.854	0.356
Diameter of the left atrium (cm)	3.5 (3.1, 3.9)	3.7 (3.3, 4.0)	2.459	0.014
Diameter of the left ventricle (cm)	4.8 (4.5, 5.3)	4.7 (4.4, 5.2)	0.889	0.374
Diameter of the right atrium (cm)	3.7 (3.5, 4.0)	3.7 (3.5, 4.0)	0.108	0.914
Diameter of the right ventricle (cm)	3.6 (3.3, 3.9)	3.6 (3.4, 3.9)	0.672	0.502
Left ventricular ejection fraction (%)	62 (60, 65)	62 (60, 65)	0.340	0.733
*Laboratory values*				
White blood cell count (× 10^9^/L)	9.8 (7.3, 12.5)	12.1 (9.0, 14.7)	3.350	0.001
Red blood cell count (× 10^12^/L)	4.2 (3.8, 4.6)	4.3 (3.9, 4.7)	1.404	0.160
Hemoglobin (g/L)	127 (113, 139)	132 (122, 142)	1.831	0.067
Platelet count (× 10^9^/L)	160 (127, 208)	149 (119, 181)	1.901	0.057
Serum creatinine (μmol/L)	78.0 (65.2, 109.0)	83.0 (69.0, 115.5)	2.840	0.005
Serum albumin (g/L)	37.8 (35.0, 40.9)	37.8 (33.6, 40.5)	0.497	0.619
Serum globulin (g/L)	25.6 (22.9, 28.3)	25.6 (22.0, 28.3)	0.316	0.752
*Operative variables*				
Surgical types			0.080	0.999
Isolated AADS	286 (65.4)	36 (65.5)		
Combined valve surgery	98 (22.4)	12 (21.8)		
Combined coronary artery bypass grafting	23 (5.3)	3 (5.5)		
Combined valve and coronary surgery	24 (5.5)	3 (5.5)		
Combined other types of cardiac surgery	6 (1.2)	1 (0.2)		
Deep hypothermic circulatory arrest	257 (58.8)	33 (60.0)	0.029	0.866
Cardiopulmonary bypass time (minutes)	209 (174, 256)	229 (196, 264)	2.025	0.043
Aortic cross clamp time (minutes)	120 (96, 147)	121 (107, 148)	1.487	0.137
Transfusion of red blood cells (units)	5 (4, 7)	7 (5, 9)	5.257	< 0.001
Transfusion of platelet (units)	3 (2, 4)	4 (3, 7)	5.009	< 0.001

AADS, Stanford type A acute aortic dissection surgery; POT, postoperative tracheostomy

**Table 2:** Multivariate analysis of independent risk factors for POT after AADS

Characteristic	Coefficient	Standard error	OR (95% CI)	*P* value
Age (years)	0.035	0.015	1.035 (1.005–1.067)	0.023
Cerebrovascular disease	0.719	0.364	2.053 (1.007–4.187)	0.048
White blood cell count (× 10^9^/L)	0.111	0.042	1.118 (1.030–1.213)	0.007
Renal insufficiency	0.874	0.333	2.396 (1.247–4.604)	0.009
Transfusion of red blood cell (units)	0.189	0.090	1.208 (1.012–1.442)	0.036
Transfusion of platelet (units)	0.166	0.071	1.180 (1.028–1.356)	0.019
Intercept	-7.466	1.174	0.001	< 0.001

AADS, Stanford type A acute aortic dissection surgery; CI, confidence interval; OR, odds ratio; POT, postoperative tracheostomy

**Fig. 3:**
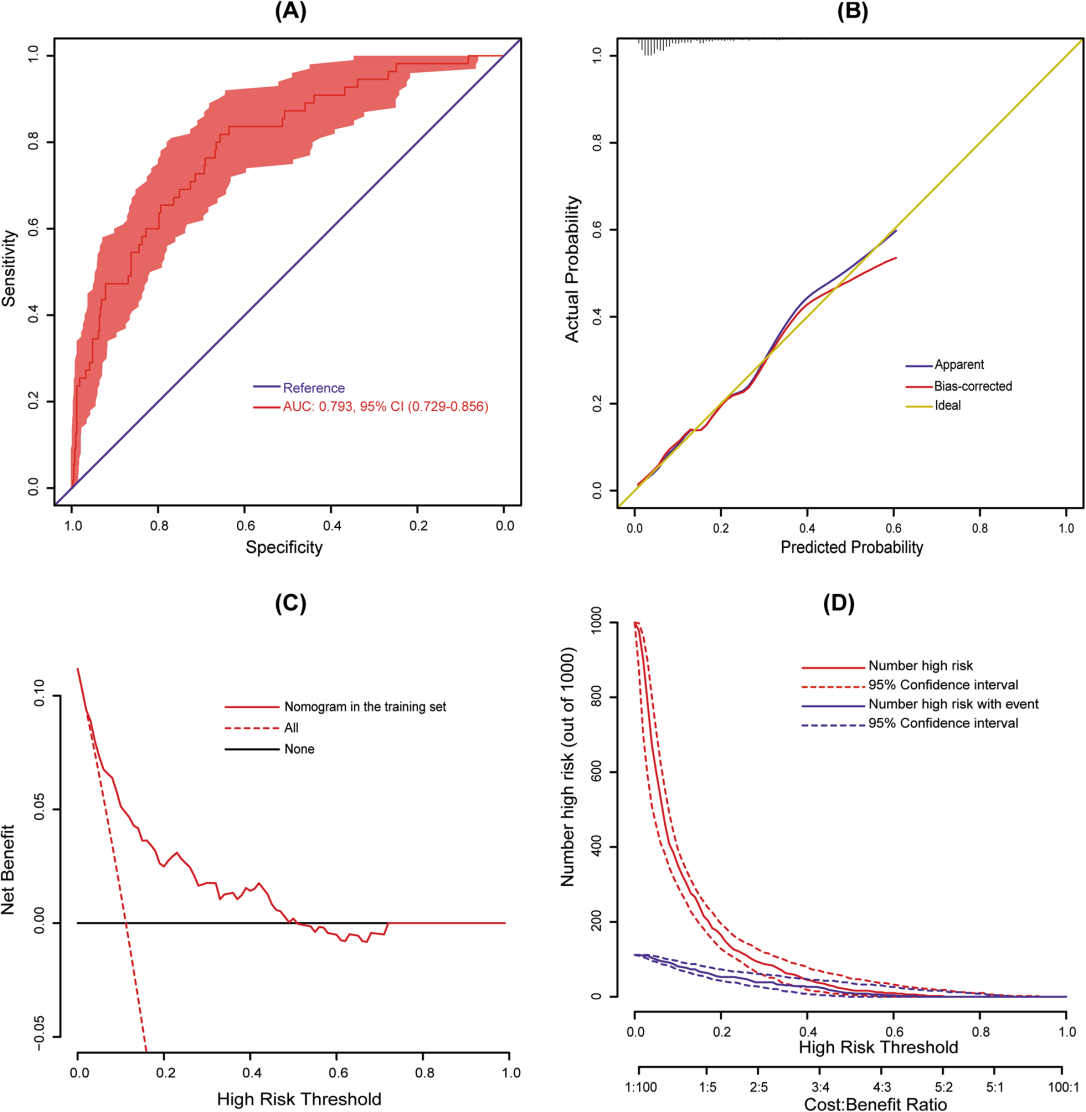
Assessment and validation of the nomogram. **a** ROC curve for the nomogram; **b** calibration plot of the nomogram. Ideal line represents perfect prediction that nomogram-predicted probability matches actually observed probability. **c** decision curves of the nomogram. Net benefit is plotted against various probability threshold; and **d** clinical impact curves of the nomogram. The total number of high-risk patients and the number of those with positive event are drawn against various risk threshold. ROC, receiver operating characteristic; AUC, area under the receiver operating characteristic curve; CI, confidence interval

The constructed model was validated internally, and the internal validation was conducted using a bootstrap method with 1,000 resamples in our cohort. The AUC was 0.793 (95% CI 0.729–0.856), indicating good discrimination of our nomogram (Fig. 3a). The calibration plot was used to test goodness-of-fit of the model, which was well calibrated through visual inspection (Fig. 3b). The decision curve demonstrated that compared with “intervention for all” or “no intervention” strategies, our predictive model could gain more clinical net benefits when the risk threshold was set between 0.04 and 0.50 (Fig. 3c). The clinical impact curve also showed that the model had good predictive ability and clinical utility (Fig. 3d).

### Risk stratification

**Fig. 4:**
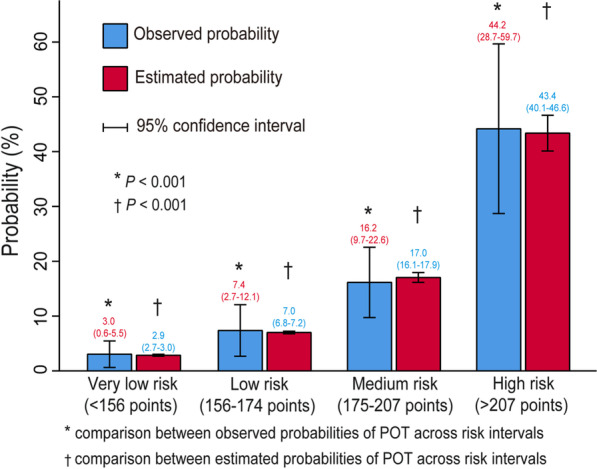
Bar chart showing the agreement between observed and estimated probabilities

Four intervals were identified as high risk, medium, low, very low risk intervals for POT based on the total calculated scores, corresponding to estimated probabilities of 0.05, 0.1, and 0.3 (Table 3). About 8.7% patients were grouped into high risk interval with a score > 207 points. Meanwhile, 197 patients (40%) were grouped into very low risk interval with a score < 156 points. On one hand, there was no significance between the observed probability and the estimated probability in each interval, showing good consistency (Fig. 4). On the other hand, the significant differences existed between the observed probabilities or the estimated probabilities in different intervals, indicating reasonable grouping.

**Table 3:** Risk intervals of POT based on the nomogram

Risk intervals	Very low risk (< 156 points)	Low risk (156–174 points)	Medium risk (175–207 points)	High risk (> 207 points)
Estimated probability (%)	< 5	5–10	10–30	> 30
Observed probability, % (95% CI)	3.0 (0.6–5.5)	7.4 (2.7–12.1)	16.2 (9.7–22.6)	44.2 (28.7–59.7)
No. of patients (%)	197 (40.0)	122 (24.8)	130 (26.5)	43 (8.7)

CI, confidence interval; POH, postoperative tracheostomy

### Outcomes

Among the whole patients, 55 patients had a tracheostomy after AADS. Compared with that of patients without POT, the proportion of patients with POT who experienced readmission to the ICU or in-hospital death after surgery was significantly larger (P < 0.001, Table 4). Significant prolongation in ICU and hospital stays were also observed in patients receiving a tracheostomy (P < 0.05). Further propensity matching analysis was performed, and 50 patients were extracted from each group (Table 5). Except for readmission to ICU, tracheostomy was still significantly related to other adverse results.

**Table 4:** Outcomes in patients with and without POT after AADS

Variables	All patients n = 492 (%)	Without POTn = 437 (%)	With POTn = 55 (%)	χ^2^/Z	*P* value
Readmission to ICU	44 (8.9)	30 (6.9)	14 (25.5)	20.731	< 0.001
ICU stay (days)	7 (5, 11)	6 (5, 9)	21 (17, 27)	11.445	< 0.001
Hospital stay (days)	21 (17, 27)	21 (16, 26)	30 (24, 42)	6.521	0.049
Mortality	49 (10.1)	29 (6.6)	20 (36.4)	48.142	< 0.001

AADS, Stanford type A acute aortic dissection surgery; ICU, intensive care unit; POT, postoperative tracheostomy

**Table 5:** Outcomes in patients with and without POT in patients undergoing AADS after propensity score matching

Variables	Included patients n = 100 (%)	Without POT n = 50 (%)	With POT n = 50 (%)	χ^2^/Z	*P* value
Readmission to ICU	19 (19)	8 (16)	11 (22)	0.585	0.444
ICU stay (days)	15 (8, 21)	8 (5, 11)	20 (17, 26)	7.535	< 0.001
Hospital stay (days)	27 (19, 36)	22 (18, 34)	29 (23, 38)	2.828	0.049
Mortality	30 (30)	10 (20)	20 (40)	4.762	0.029

AADS, Stanford type A acute aortic dissection surgery; ICU, intensive care unit; POT, postoperative tracheostomy

## Discussion

This study identified a rate of POT (11.2%) in patients undergoing AADS, which was similar to previously reported data [5, 10]. In addition, it may not be surprising that we found patients with a tracheostomy had increased resource utilization or in-hospital mortality following AADS compared to those without a tracheostomy. In-hospital mortality among patients treated with tracheostomies after AADS was 36.4%, which was comparable with previous results of cardiac surgical patients [11]. These findings in our study provide insight on the outcomes of the patients requiring tracheostomy after AADS, and emphasize the urgent need for early identification of these high-risk populations requiring tracheostomy.

Our study is the first to investigate the predictors of POT in patients with AADS and to build a nomogram model to predict POT. Based on the clinical data of 492 patients who underwent AADS at one institution, preoperative factors including age, cerebrovascular disease, WBC, renal insufficiency, as well as intraoperative factors including RBC and platelet transfusion, were identified to be independently associated with POT after AADS. Our predictive model was established relying on these risk factors and was validated with well predictive performance and good clinical utility. In order to better apply the model to the clinic, we further defined four levels of risk intervals to facilitate the risk stratification of patients.

The preoperative cerebrovascular disease was the risk factor (OR > 2) that contributed more to the development of requiring POT in our study. Mukerji et al. found that prior cerebrovascular disease was significantly related to increased intubation duration and lower successful extubation rate [12]. Stroke patients were at high risk of respiratory and swallowing dysfunction, and about a quarter of them required ventilatory support [13]. However, iatrogenic laryngotracheal stenosis which was commonly caused by endotracheal and/or tracheostomy tubes injury presented with a higher risk of tracheostomy dependence [14]. Our analysis also revealed the pre-existing renal insufficiency as another associated risk factor. Songdechakraiwut et al. reported that chronic renal insufficiency had 2.85-fold increased odds of POT after aortic aneurysm repair [15]. In addition, preoperative renal insufficiency has been indicated to be an important predictor of respiratory complications by Etz et al. [16]. The observed impairment of respiratory muscle strength and endurance in patients with chronic renal failure might predispose them to respiratory muscle fatigue, which may partly explain the potential association between renal insufficiency and the need for tracheostomy [17].

It was shown that the older age was correlated with increased risk of POT among our target study population, but the results of whether the probability of tracheostomy increases with age in several studies were inconsistent [8, 18, 19], and we think that this difference may be partly due to the different disease populations studied. Suzuki et al. suggested that age was negatively correlated with elevated WBC count before AADS [20]. Interestingly, our study showed that the higher the preoperative WBC count, the greater the possibility of necessitating tracheostomy after AADS. Ge et al. have identified elevated WBC count as independent risk factor for prolonged mechanical ventilation in patients undergoing AADS [21]. In addition, WBC elevation is a well-known acute-phase systemic inflammatory response in patients with aortic dissection, and it was also found to be an independent predictor of in-hospital death [20, 22]. Although the mechanism of the link between elevated WBC count and requiring POT is unclear, we speculate that it may be in relation to inflammatory damage involving the lungs induced by the acute aortic dissection. The strategy of leukocyte depletion during CPB which attenuates the CPB-induced inflammatory response by reducing endothelial activation and neutrophil transmigration can lead to improved oxygenation, earlier extubation, and shorter hospital stays [23, 24].

Previous study has suggested that a lower preoperative platelet count was a risk factor for prolonged mechanical ventilation or postoperative pneumonia [2, 25]. But the preoperative platelets of the patients who underwent POT in our study were not significantly lower. Zindovic et al. confirmed that aortic dissection caused significant activation of the coagulation system including platelet activation, while surgery caused further derangement of the hemostatic system [26], and AADS is often complicated by excessive bleeding and allogeneic blood products transfusion [27]. In our analysis, platelet transfusion was identified as an intraoperative predictor for POT, consistent with previous studies [15], they reported that more intraoperative transfusion of platelet was independently associated with increased risk of POT with an odds ratio of 1.04. Prophylactic or emergency platelets transfusion is usually used in cases of a reduction in platelet count or dysregulation in platelet function which is related to bleeding risks and hemorrhagic complications [28]. However, patients receiving platelet transfusion during surgery experienced prolonged ventilation and intensive care after cardiac surgery [29]. Kornblith et al. also indicated that platelet transfusion was a significant independent risk factor for acute respiratory distress syndrome [30]. The potential explanations for these connections may come from conclusions of some published studies, that is, platelets play an important role in the pathogenesis of acute lung injury, and the platelet transfusion-related acute lung injury was the main determinant of increased mortality [31, 32]. Nonetheless, some measures may inhibit the release of proinflammatory cytokines, reduce reperfusion lung injury and preserve lung function, such as ulinastatin administration, a urinary trypsin inhibitor during CPB [33].

Transfusions of RBC and platelets have been related to systemic inflammatory response syndrome, transfusion related acute lung injury, and mortality, leading to increased hospitalization and healthcare costs [34]. In this study, intraoperative RBC transfusion was correlated with a dose-dependent increase in the risk of POT after AADS, although RBC transfusions are used to treat hemorrhage and to improve oxygen delivery to tissues [35]. A restrictive strategy of RBC transfusion has been found to be safe in patients undergoing some surgeries of high-risk large blood loss [36–38]. The use of intraoperative autologous platelet rich plasma in patients undergoing AADS was also related to a reduction in intraoperative RBC transfusions, as well as reduced ventilation time [39]. In addition, the application of moderate hypothermic circulatory arrest and unilateral selective antegrade cerebral perfusion (MHCA/uSACP) during the AADS was shown to represent a protective factor for POT [5].

The benefits of POT may be improved by the optimal timing of it for cardiac surgical patients, whereas this optimal time point is still unclear and controversial [4, 40]. Nonetheless, our study found that the relationship between needing POT and poor outcomes was still significant after limiting any influence of selection bias and the heterogeneity in preoperative variables and surgical extent. Consideration should be given to reducing unnecessary tracheostomy for the low-risk patients needing tracheostomy after AADS. Of course, our model may help early management of high-risk patients in order to reduce the occurrence of POT, including increasing awareness of implementing more careful postoperative airway care.

Some limitations are important to acknowledge. Inasmuch as this is a single-center study with a small sample size, our predictive model may not necessarily be applicable to other centers. Meanwhile, due to the retrospective nature of the study and lacking external validation, caution must be exercised before applying our model prospectively. Additionally, the implementation of the tracheostomy was determined by the cardiac intensivists at that time, which may induce bias as well. Moreover, our model involves blood transfusion variables, but we failed to collect data such as the amount of blood lost, hemoglobin, platelet count, etc. during the operation.

## Conclusion

The incidence of requiring tracheostomy among the population with AADS was 11.2% in this study. Requirement for a tracheostomy in patients after AADS was associated with significant prolonged ICU and hospital stays and increased mortality. Our study identified six significant risk factors for POT and established a new model for predicting requiring POT in patients undergoing AADS. The constructed model may enable guidance of proper prevention at the early stage and better estimation of need to perform a tracheostomy in the follow-up treatment after AADS.

## Data Availability

The datasets generated and/or analysed during the current study are not publicly available because it is part of a large comprehensive study but are available from the corresponding author on reasonable request.

## Abbreviations

AADS: Stanford type A acute aortic dissection surgery
AUC: Area under the receiver operating characteristic curve
CI: Confidence interval
CPB: Cardiopulmonary bypass
ICU: Intensive care unit
MHCA/uSACP: Moderate hypothermic circulatory arrest and unilateral selective antegrade cerebral perfusion
OR: Odds ratio
POT: Postoperative tracheostomy
ROC: Receiver operating characteristic
RBC: Red blood cell
WBC: White blood cell
